# Trade-offs in cavefish sensory capacity

**DOI:** 10.1186/1741-7007-11-5

**Published:** 2013-01-24

**Authors:** Helen Gunter, Axel Meyer

**Affiliations:** 1 Lehrstuhl für Zoologie und Evolutionsbiologie, Department of Biology, University of Konstanz, Universitätsstrasse 10, 78457 Konstanz, Germany; 2 Zukunftskolleg, University of Konstanz, Universitätsstrasse 10, 78457 Konstanz, Germany

## Abstract

In caves one repeatedly finds strikingly convergent patterns of evolution in diverse sets of organisms involving 'regressive' traits such as the loss of eyes and pigmentation. Ongoing debate centers around whether these regressive traits arise as the result of neutral evolutionary processes, or rather by natural selection of 'constructive' traits that arise at the expense of eyes and pigmentation. Recent research on cavefish points to the latter, suggesting that the 'constructive' trait vibrational attractive behavior and the reduction of eye size may share a common genetic basis.

See research article http://www.biomedcentral.com/1741-7007/10/108

## Commentary

The perpetually dark, nutrient-poor environments inside caves are both novel and stressful, driving the convergent evolution of 'cave-specific' characters in diverse lineages. Cave dwelling species have long been studied by evolutionary biologists, not because of their adaptive characters, but rather for regressive characteristics, which include the loss of pigmentation and eyes. Even in the *Origin of Species*, Darwin pondered why evolution would favor regressive characters in these lineages, reasoning that eyes should at least be of little harm to cave organisms [1]. Neutral processes or drift were later considered to be causing these reductions, whereby mutations that lead to the development of only rudimentary, non-functional eyes were no longer selected against in lightless cave environments. Their loss would presumably save energy that might be re-allocated to other structures that enhance survival and reproduction in these nutrient-poor environments.

However, recent research - exemplified by the Mexican cavefish (*Astyanax mexicanus*) - points rather to the evolution of 'constructive' (without wanting to imply teleological evolution), useful characters that arise, possibly at the expense of adaptations for life on the surface [2]. For example, in addition to their loss of eyes and pigmentation, cavefish display adaptive sensory characters that may promote their survival, such as an increased number of taste buds, larger olfactory bulbs and hypothalamus and larger numbers of neuromasts, cells located in the skin that act as a kind of long distance touch [3]. Understanding whether such regressive and adaptive traits evolve in parallel as a result of pleiotropy or are due to entirely independent processes is highly interesting and hotly debated [3,4].

Cavefish (*A. mexicanus*) are a favorite model for studying evolution in caves as this species includes 29 separate populations in Northern Mexico that display convergent (but non-identical) cave phenotypes [4,5]. Importantly, genetic studies can easily be performed with this species, as the cave populations remain completely interfertile with their surface-dwelling relatives in spite of their dramatic morphological differences. The molecular basis of eye loss in cavefish has already been the topic of intensive research, which has shown that overexpression of sonic hedgehog (*shh*) along the embryonic midline leads to apoptosis and resorption of the developing eyes. Interestingly, *shh *overexpression simultaneously causes an increase in the number of taste buds and expansion of the forebrain and hypothalamus, which may lead to improved prey capture ability in the dark cave environment. Taken together, this suggests that natural selection favors sensory expansion, with the secondary loss of eyes occurring as a result of pleiotropic processes [6].

Given the strong phenocopying of the cavefish morphology induced by *shh *overexpression, it was expected that quantitative trait loci (QTL) studies would point to *shh *as a causative mutation for cavefish eye reduction. However, in spite of 12 QTLs being identified for eye size, none of them were located even in the vicinity of *shh *[7]. Therefore, *shh *may operate up- or downstream of the causative mutation for eye loss, suggesting that further work is required to determine whether eye loss occurs due to pleiotropy.

This led Yoshizawa *et al. *[2] to ask whether an alternative adaptive trait may be linked to eye loss in cavefish. Vibration attractive behavior (VAB), defined as the swimming of a cavefish towards oscillating objects in water, is a potentially adaptive trait that has evolved repeatedly in cave populations. Surface fish rarely display this behavior as it attracts the attention of predators; however, in the context of a lightless cave environment, where starvation is a far bigger threat, this behavior is likely to be adaptive as it aids in catching insects that fall on the surface of the water [8]. Previous research has demonstrated that superficial neuromasts (SNs) of the anterior lateral line organ sense these surface vibrations - sort of a long-distance touch mechanism [8]. Thus, Yoshizawa *et al. *[2] sought to determine the genetic basis of eye size, VAB and SN number, to determine whether they are likely to be linked.

First, they conducted fine scale morphological and functional studies, which identified that SNs, specifically in the vicinity of the eye orbit (EO SNs), play a strong functional role in VAB and that the number of EO SNs is negatively correlated with eye size. Second, genetic analyses identified QTLs for eye size, VAB and EO SN number. Importantly, QTLs for VAB, EO SNs and eye size are located in overlapping regions of the genome, suggesting that these traits may, in part, share a common genetic basis. This indicates that the likely adaptive behavioral trait (VAB), enabled through enhanced EO SNs, arises at the expense of eye size through genomic regions that are in close vicinity to one another. They also convincingly showed that eye degradation alone (induced by *shh *over-expression) is not sufficient to induce VAB or an increase in EO SNs. This demonstrates that eye degradation and the concomitant gain of VAB are not the result of *shh *overexpression, lending further support to previous results suggesting that the causative mutation for the cavefish phenotype does not reside in *shh*.

The highly integrative approach adopted by Yoshizawa *et al. *[2], incorporating genetics, morphology and behavioral and developmental biology, has propelled the regressive/constructive debate of cave adaptations into a new direction. Contrary to this study, previous investigations have not detected a correlation between free neuromast number and eye orbit size [3]. The more detailed methods employed in this study that distinguished between SNs of the suborbital bone and in the eye orbit enabled Yoshizawa *et al. *[2] to highlight the most functionally important SNs, and led to the detection of a strong positive correlation between these traits. Moreover, functional analyses confirmed that EO SNs are essential for VAB, despite representing only a small proportion of the total SNs in the anterior lateral line apparatus.

The observation of fine-scale regional functionalization within the lateral line echoes recent results from studies on sticklebacks demonstrating that the lateral line is patterned by multiple independent genetic modules, which may facilitate rapid and significant sensory diversification [9]. The results of this study run counter to previous research by Wilkens *et al*., in which exhaustive hybrid crosses between cave and surface populations displayed no correlations between a large number of 'constructive' and regressive traits [3]. However, overlapping QTLs for multiple regressive and 'constructive' traits have been identified by Protas *et al. *[7], suggesting that this is not a simple matter.

Is the debate of whether these convergent traits are the result of independent or pleiotropic genes resolved? Yoshizawa *et al. *[2] do present a compelling demonstration of an association between eye size and EO SN number, but it still falls short of demonstrating pleiotropy of 'constructive' and regressive genes. One (likely) scenario that is not assessed by this study is that the overlapping QTLs may be the result of tight linkage rather than genuine pleiotropy - it is impossible to distinguish these two possibilities when the causative mutation has not been identified. Moreover, the regressive phenotypes of the different cavefish populations are most likely caused by several independent mutations as crosses between some cavefish populations result in offspring that more closely resemble surface relatives than cavefish [4]. Finally, as VAB is a complex trait that can be achieved through multiple morphological bases and follows very different patterns of inheritance in separate cavefish populations, it is plausible that constructive traits like VAB are pleiotropic to regressive traits in some populations but not others [10]. In order to test for pleiotropy empirically, future research should focus on isolating the causative mutations that underlie both eye size and enhanced numbers of neuromasts in the eye orbit region.

Studying the genetic basis of sensory evolution in cavefish may provide insights into their patterns of diversification as sensory capacity may act to reinforce reproductive isolation between cave and surface populations, thus enabling speciation in the face of introgression. For example VAB and SN number and size are controlled by paternally inherited genes for the Pachon cave population, such that hybrid offspring of surface males and Pachon females do not display VAB, but offspring of Pachon males and surface females do [10]. As VAB is essential for survival in a darkened environment [8], Pachon-surface hybrids would, on average, display significantly reduced fitness, thus promoting reproductive isolation.

Yoshizawa *et al. *[2] make an exciting contribution to our understanding of the relationship between 'constructive' and regressive traits in a model cave-dwelling organism. While their study does not conclusively demonstrate a pleiotropic basis for the highlighted 'constructive' and regressive traits, they do break new ground in demonstrating a strong correlation between two such traits, which are encoded by overlapping genomic regions. We are living in an exciting time, where the utilization of interdisciplinary techniques can shed light on questions, such as the evolutionary origin of regressive traits, that have perplexed biologists ever since Darwin.
